# Ultrasensitive detection of serum hepatitis B virus by coupling ultrafiltration DNA extraction with real-time PCR

**DOI:** 10.1371/journal.pone.0170290

**Published:** 2017-02-09

**Authors:** Bin Wu, Feng Xiao, Peiwen Li, Yan Du, Jinqiong Lin, Kaihua Ming, Bin Chen, Xiuxia Lei, Banglao Xu, Dayu Liu

**Affiliations:** 1 Department of Laboratory Medicine, Guangzhou First People’s Hospital, Affiliated Hospital of Guangzhou Medical University, Guangzhou, China; 2 Clinical Molecular Medicine and Molecular Diagnosis Key Laboratory of Guangdong Province, Guangzhou, China; Centre de Recherche en Cancerologie de Lyon, FRANCE

## Abstract

**Background:**

A simple and reliable DNA extraction of hepatitis B virus (HBV) is critical in developing an ultrasensitive detection method for HBV infection. Current commercially available serum Hepatitis B Virus (HBV) DNA extraction methods are time-consuming, expensive and/or require specialized equipment, which hinders wide adoption of clinical laboratories. This study offers a report on an ultrasensitive HBV DNA detection method by coupling serum HBV DNA extraction by ultrafiltration (UF) with real-time PCR (qPCR) detection.

**Methods:**

Serum proteins were precipitated by phenol to release HBV DNA in the supernatant which was then transferred to the UF devices. The resultant DNA concentrate was eluted and released into qPCR pre-mixture. The UF-qPCR assay performance, including recovery rate, linearity, detection sensitivity, precision and diagnostic accuracy that compared to the CAP-CTM V2.0 assay by analyzing batched low viral load clinical samples was evaluated.

**Results:**

The recovery rate of the UF-based HBV DNA extraction method was above 80%. The assay linearity was demonstrated with a slope of 0.95 and R^2^ values of 0.99. Limit-of-detection (LOD) of the UF-qPCR assay was determined to be 12.1IU/ml. The coefficient of variation (CV) of HBV quantitation for high, low and limit titer samples was 2.28%, 5.77% and 25.59%, respectively. Accuracy of the UF-qPCR assay was confirmed with the reference panel, and there was a strong correlation between these two methods (R^2^ = 0.55, *p* < 0.01).

**Conclusions:**

The UF-qPCR assay is reliable, highly sensitive, affordable and time-saving, and the method can be used for ultrasensitive detection of serum HBV.

## Introduction

Hepatitis B Virus (HBV) infection is one of the most common causes of liver diseases ranging from acute hepatitis to chronic hepatitis, liver cirrhosis, and hepatocellular carcinoma (HCC) [1]. Over 2 billion people throughout the world have been infected with HBV and more than 350 million of them are chronically infected carriers [2]. The virological diagnosis and monitoring of the HBV infection are based on immunoassays detecting viral antigens and specific anti-HBV antibodies as well as nucleic acid detection assays targeting genomic material of the virus [3]. The presence of HBV DNA in peripheral blood is a reliable marker of active HBV replication. In comparison with immunoassays, HBV DNA detection and quantification are more useful in the diagnosis of infection, therapeutic decision-making, and assessment of the response to therapy [4]. In addition, HBV DNA quantitation can be used to monitor viral replication kinetics in order to better understand the mechanisms of infection and the virologic response to antiviral therapy. To date, a series of HBV DNA detection assays have been developed, including spot hybridization technique [5], quantitative real-time PCR (qPCR) [6], DNA biosensor [7], loop-mediated isothermal amplification assay (LAMP) [8], rolling circle amplification (RCA) [9], et al. Among these assay systems, qPCR is strongly recommended by current consensus guidelines due to its sensitivity, specificity, accuracy and broad dynamic range [10]. The serum HBV DNA detection system using sensitive and accurate qPCR assays is crucial to predict the response to therapy, to determine therapy initiation, to monitor resistance to therapy, to establish treatment success, and to evaluate the risk factors for cirrhosis and the progression of HCC [11,12].

Serum HBV DNA levels are unstable over time and are dependent upon the infection phase: the immunotolerant phase after acute infection is characterized by high levels of viral replication as well as generally lower, often fluctuating HBV DNA. The “clinical cure” phase is characterized by persistent undetectable levels of viral replication, which usually confers an excellent prognosis. During the reactivation phase, the viral load generally recurs to high levels [13]. The decision of clinical cure is dependent on the sensitivity of the assay used [10]. The guidelines for the prevention and treatment of patients with HBV infection issued by World Health Organization (WHO) [14] revealed that the undetectable viral load of HBV DNA for qPCR assays is a concentration below 15 IU/ml. However, most of the currently used qPCR assays for analyzing HBV DNA cannot meet this requirement; thus, it is unable to detect the presence of such low levels of HBV DNA. This insufficient detection sensitivity often leads to misjudgment of disease curability and is accompanied by an early termination of antiviral therapy [15]. As reported by Liang Y et al. [16], early discontinuation of antiviral therapy is typically associated with virological relapse. In order to overcome the limitation in detection sensitivity, a series of ultrasensitive qPCR assays were developed [17,18]. These ultrasensitive assays significantly improved HBV DNA detection sensitivity by applying increased sample input volumes [19]. For example, the CAP-CTM assay (Roche) can reach a limit-of-detection (LOD) down to12 IU/ml with a sample input volume of 500 μl [20]. The application of ultrasensitive HBV qPCR assays allows detection of viral breakthrough at an early stage as well as discontinuation of antiviral therapy at a proper time, thus improved the control of patients with low viral load [18]. However, the wide adoption of current ultrasensitive HBV quantitation assays in clinical laboratories faces challenges due to the time-consuming nature of the serum HBV DNA extraction as well as expensive costs of specialized equipment [21]. Therefore, developing a simple, rapid and cost-effective DNA extraction method is of particular importance for ultrasensitive HBV DNA detection.

Ultrafiltration (UF) is a variety of membrane filtration in which forces similar to pressure or concentration gradients lead to a separation through a semipermeable membrane. The suspended solids and solutes of high molecular weight are retained in the so-called retentate while water and low molecular weight solutes pass through the membrane in the permeate. UF membranes are defined by Nominal Molecular Weight Limit (NMWL, typically reported in daltons) of the membrane used. UF is based on size exclusion or particle capture, enabling these filters to concentrate large volumes of sample in a short time [22]. UF has been wildly used in biomolecular concentrating, water treatment and food processing [23,24]. This approach has also been reported to hold the potential for nucleic acid concentration and purification. Hancher and Ryon [25] first reported RNA purification by UF through polymeric UF membranes. Additionally, Hirasaki et al. [26] examined the DNA filtration through membranes with mean pore diameters of 15 and 35 nm and found the permeability of DNA molecules decreased with an increase in DNA size. In addition, the deformability, chemical structure and geometrical size of DNA molecule also impact the sieving effect. Arribas et al. [27] reported that the DNA concentration by UF improved detection sensitivity of Epstein-Barr Virus DNA in cerebrospinal fluid samples. In comparison to other nucleic acid preparation technologies, UF possesses the following advantages: simple procedures, low cost, and the ability to handle large-volume sample. Therefore, UF is a promising method of DNA preparation for ultrasensitive HBV DNA detection.

Although UF is useful of its ability to handle large-volume sample, the direct extraction of DNA from serum samples by UF remains challenging due to the complexity of serum components, especially high levels of protein. In order to overcome this limitation, the present study developed a UF-based DNA extraction method that includes sample preparation, ultrafiltration and elution. The serum proteins were first precipitated by phenol, and the supernatant with released HBV DNA was then filtrated using the UF device. Finally, the DNA concentrate was eluted and released into a qPCR pre-mixture. The performance characteristics of the UF-qPCR assay were evaluated and demonstrated high DNA recoveries with good reproducibility. These data demonstrate that the UF-qPCR assay can be used in ultrasensitive detection of HBV DNA. And the current method also gives new insight on the application of ultrafiltration of blood samples with low DNA content.

## Methods

### Ethics statement

This study was approved by the Ethics Committee of Guangzhou First Peoples’ Hospital in accordance with the Helsinki Declaration. All participants gave written informed consent before taking part in the study.

### Samples

The peripheral blood samples from patients infected with HBV were collected between September and December in 2015 at Guangzhou First Peoples’ Hospital, Guangzhou Eighth Peoples’ Hospital and The First Affiliated Hospital of Sun Yat-Sen University. All samples were stored at -20°C until use. Forty of the samples were analyzed with CAP-CTM V2.0 and UF-qPCR in parallel.

### Extraction of serum HBV DNA by UF

**Fig 1:**
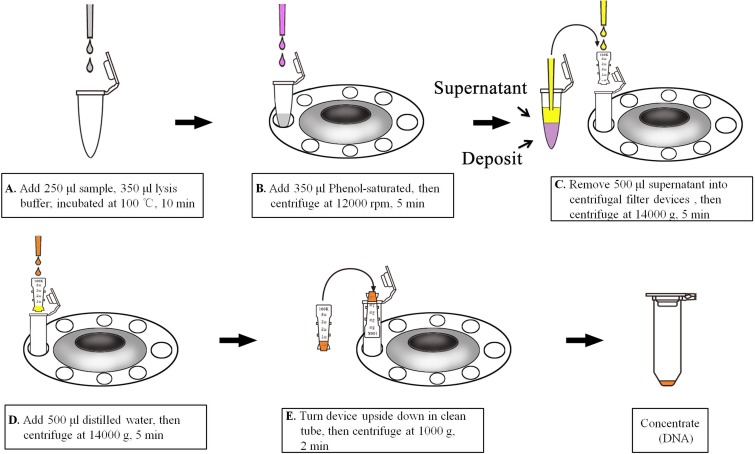
Illustration of HBV DNA extraction by UF.

As illustrated in Fig 1, 250 μl of serum sample or HBV Quantitation Standard (QS) (Daan Gene, Guangzhou, China) was mixed with 350 μl of lysis buffer (Daan Gene), and the mixture was incubated at 100°C for 10 min. Subsequently, 300 μl of phenol-saturated water (pH 7.9; Sangon Biotech, Shanghai, China) was added to the lysate. The mixture was kept at room temperature for 5 min, followed by centrifugation (12000 rpm, room temperature, 5 min) in order to release DNA into (about 500–550 μl) the supernatant. Next, the supernatant (~500 μl) containing free DNA was transferred into a 0.5 ml Amicon Ultra centrifugal filter device (Millipore, USA) and centrifuged at 14000 g for 5 min. After washing with 500 μl of distilled water, the UF device was kept upside down, and DNA was eluted in a clean tube by centrifuging at 1000 g for 2 min.

The extent of HBV DNA adsorption to the filter devices was examined as described previously with a little modification [28]. Briefly, 300 μl of purified HBV DNA of 20, 100 and 200 ng/μl were added into filter devices with different NWMLs (30K, 50K and 100K). The filter devices were kept at room temperature for 1h before the samples were reclaimed. The DNA concentrations were determined before and after the incubation using a Nanodrop 2000 spectrophotometer (Thermo Scientific, USA). The amount of DNA detained on the UF device was estimated by the difference in DNA concentration and volumes.

The quality of HBV DNA extracted by the UF and conventional methods was evaluated by OD_260_/_280_ ratio. HBV DNA was prepared using various protocols that included boiling extraction (with lysis buffer purchased from Daan Gene), magnetic beads extraction (with MagicMag Genomic DNA Micro Kit, Sangon Biotech) and column extraction (Column Virus Genomic DNA Isolation Kit, Sangon Biotech). OD_260_/_280_ ratio of the DNA concentrate was determined using the Nanodrop 2000 spectrophotometer.

### Real-Time PCR

The Real-Time HBV PCR assay with a commercial kit (catalog number: DA-B051, Daan Gene) was performed according to the manufacturer's instructions. The final volume was 30 μl, containing 20 μl qPCR pre-mixture and 10 μl concentrated DNA sample. The qPCR reactions were performed on a Real-Time PCR System (ABI 7500, Applied Biosystems). The cycling parameters consisted of 2 min incubation at 93°C in order to activate the Taq polymerase, 10 cycles of 93°C for 45 s and 55°C for 60 s, followed by 30 cycles of 93°C for 30 s and 55°C for 45 s.

In parallel, the CAP-CTM V2.0 assay was performed on a COBAS TaqMan Analyzer. After the automated sample preparation on the COBAS AmpliPrep Instrument, processed specimens, including HBV DNA and HBV QS DNA, were added to the PCR pre-mixture. The qPCR assay included the HBV QS (serving as an internal reference) test to compensate for the effects of inhibition as well as to control the preparation and amplification processes. The quantitative results are reported in international units (IU) per milliliter.

### Establishment of standard curves

For standardization of the quantitative HBV DNA detection assays, the HBV QS serum samples with known copy numbers (2.30, 3.30, 4.30, 5.30, 6.30 log_10_ IU/ml) were used in the experiment. The HBV DNA extracted from the QS samples using centrifugal filter devices with 100K NWML was then subjected to a qPCR system. The *Ct* (Cycle threshold, *Ct*) values of individual dilutions were plotted against the initial virus copy number, which resulted in typical standard curves.

### Recovery rate of HBV DNA

The HBV DNA was extracted from QS serum samples with high (6.30 log_10_ IU/ml) and low (4.20 log_10_ IU/ml) -titer using UF devices with different NWMLs (30K, 50K and 100K), MBs and DNA-extraction column. The extracted HBV DNA was quantified using the qPCR assay. The recovery rate of HBV DNA was calculated as follows: *R = (C×V)/(C*_*0*_*×V*_*0*_*)*×100%, where *C* and *V* represent HBV DNA quantity (IU/ml) and volume of the concentrate, *C*_*0*_ and *V*_*0*_ represent HBV DNA quantity (IU/ml) and volume of the input QS serum.

### LOD

The LOD of the UF-qPCR assay was determined by analyzing the serial dilutions of a low-titer HBV QS serum sample (4.20 log_10_ IU/ml, Daan Gene).

### Linearity and precision

In order to investigate linearity, high-titer HBV clinical specimens (2.5×10^8^ IU/ml) were serially diluted in 1:10 steps to 25 IU/ml. At each level, three replicates were analyzed. The difference between the expected and the observed value was assessed.

The precision of the UF-qPCR assay was evaluated by analyzing 20 replicates of a high-titer (6.30 log_10_ IU/ml), a low-titer (3.20 log_10_ IU/ml) and a limit-titer (10 IU/ml) QS sample, respectively. CVs were calculated out from the quantitative results.

### Statistical analysis

The quantitative results were log transformed for further analysis. The continuous variables were expressed as mean and standard deviation (SD). The correlation analysis between the UF-qPCR and CAP-CTM V2.0 assays was analyzed by Pearson’s correlation and linear regression. The Bland–Altman plots were used in order to represent the degree of agreement between quantitative results obtained from UF-qPCR and CAP-CTM V2.0. All statistical analyses were performed using the graphpad prism (Graphpad Software. San Diego, CA), and *P* <0.05 was considered significant.

## Results

### Serum protein removing

**Fig 2:**
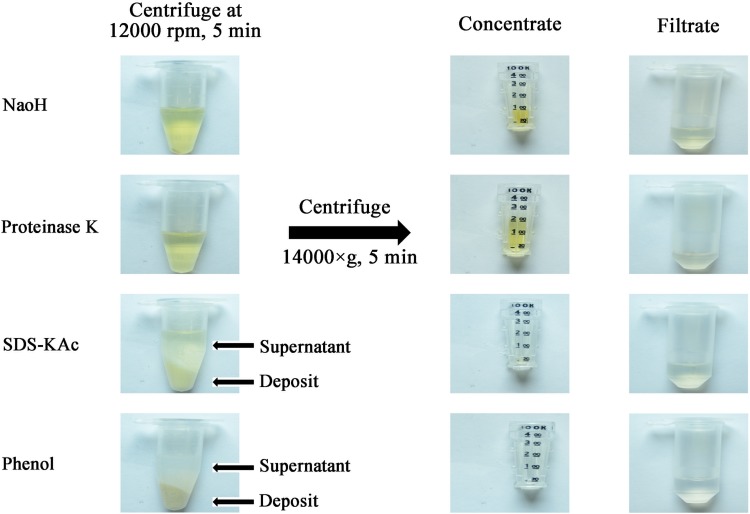
Effects of serum protein removing by different methods. Figures showing the generation of precipitant after treating the serum samples with NaOH, proteinase K, SDS-KAc and phenols, respectively (left), and concentrates obtained from the supernatants by ultrafiltration (right). NWML of the ultrafiltration device used was 100K.

Various means were tried to remove serum proteins, including proteinase K digestion and protein precipitation with NaOH, SDS-KAc and phenol. The performances of these methods were evaluated (Fig 2). Based on the results, phenol treatment was selected as the means of protein removing (operation procedure shown in Fig 1).

### Performance of UF DNA extraction

**Fig 3:**
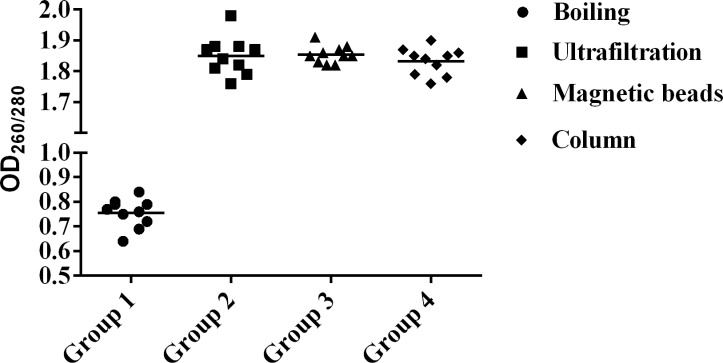
OD_260/280_ ratioof DNA samples prepared using various methods.

The optimum conditions for UF with phenol precipitated samples were determined as follows: NWML, 100K; centrifugal force, 14000 g; centrifugation time, 5 min. The OD_260_/_280_ ratio of DNA prepared by UF ranged from 1.7 to 2.0, indicating the UF method is able to provide DNA concentrate of high purity. The comparison of purity of DNA concentrates prepared using various methods were shown in Fig 3. Except for boiling, all other methods provided highly purified DNA. The qPCR with DNA concentrates prepared by UF yielded an averaged amplification efficiency of 94%, indicating the concentrates were well compatible with the PCR amplification.

**Fig 4:**
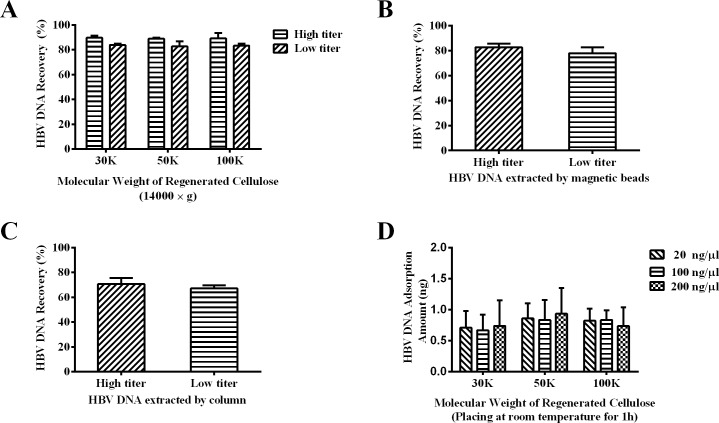
Recovery of HBV DNA using UF devices with different NWMLs, magnetic beads and column. (A) The recovery rates of high-titer (6.3 log_10_ IU/ml) and low-titer (4.2 log_10_ IU/ml) HBV samples using the UF devices of different NWMLs; (B) The recovery rates of HBV DNA obtained using magnetic beads method; (C) The recovery rates of HBV DNA obtained using the DNA-extraction column method; (D) Adsorption amount of the HBV DNA on the filter devices (30K, 50K and 100K). Samples used were purified HBV DNA of different concentration (20, 100 and 200 ng/μl). The devices were placed at room temperature for 1h before detection.

The recovery rates of HBV DNA using filtration device of different NWMLs (30K, 50K and 100K) were above 80% in both the high and the low-titer groups (Fig 4A). The recovery rates of high-titer QS samples were slightly higher than those of low-titer ones. However, the difference was not found to be statistically significant. At the same time, the recovery rates obtained with MBs were 82.6% for the high-titer sample and 77.9% for the low-titer one, and those obtained with the DNA-extraction column were 70.6% (high-titer) and 67.2% (low-titer) (Fig 4B and 4C).

The amount of HBV DNA detained on the centrifugal filter device was evaluated by solution depletion (Fig 4D). The loss of DNA was estimated by (*C*_*0*_*×V*_*0*_*-C×V)*, where *C* and *C*_*0*_isthe concentration of the recovered and the original sample, respectively; *V* and *V*_*0*_isthe volume of the recovered and the original sample, respectively. For HBV DNA samples of these groups, the changes in DNA amount were all less than 1ng, and no significant differences were found among groups.

### HBV DNA quantitation

The slopes of the standard curves were generally at or around 3.31. The consistency of the replicates was validated by a correlation coefficient (R^2^) of 0.99, which indicates the linearity of the *Ct* values plotted in the standard curves (S1 Fig). All these data were detected according to the standard curves in order to evaluate the clinical performance characteristics of the present method including precision, linearity, LOD and diagnostic accuracy.

**Fig 5:**
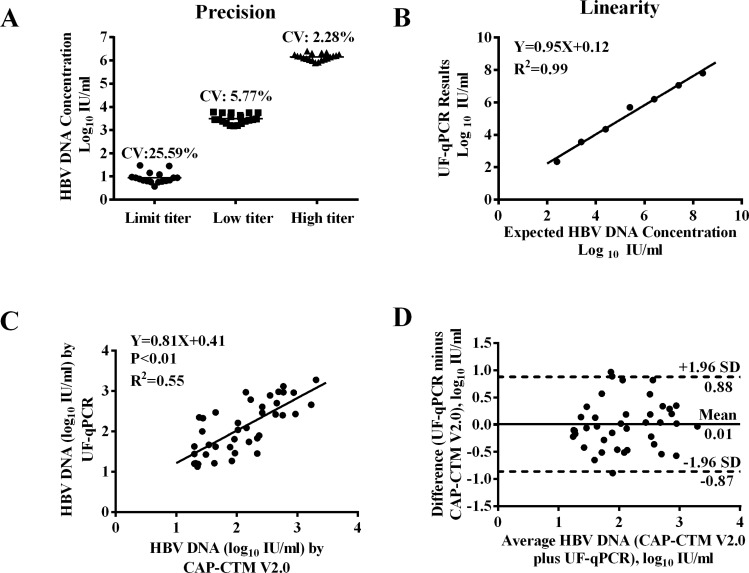
Performance of UF-qPCR assay. (A) CVs of the DNA quantitation of High-titer (6.3 log_10_ IU/ml), low-titer (3.2 log_10_ IU/ml) and limit-titer (10 IU/ml). The QS samples were treated using UF devices with 100K NWML; (B) Linearity of HBV DNA quantitations determined using the UF-qPCR assay. Samples used were serial dilutions of a high-titer HBV clinical specimen; (C) Correlation analysis of HBV DNA quantitative results (n = 40) between UF-qPCR and CAP-CTM V2.0 assay system. (D) Consistency of UF-qPCR and CAP-CTM V2.0. The difference between UF-qPCR and CAP-CTM V2.0 measurements is plotted as a function of the mean of these two values. The area between the dashed lines corresponds to the mean difference ±1.96 SD.

Reproducibility was evaluated at three levels of viral load (1.0, 4.2 and 6.3 log_10_ IU/ml). The precision (CV %) of this experiment is shown in Fig 5A. The precision of the DNA quantitation determined in these three groups was 2.28%, 5.77% and 25.59%, respectively.

Linearity was demonstrated between 25 and 2.5×10^8^ IU/ml in each assay, using triplicate measurements of the serial dilutions of a high-titer sample. The assay linearity was assessed as the difference between the detected values and the nominal values assigned to the samples. As shown in Fig 5B, the linear regression equation was Y = 0.95X + 0.12, with a correlation coefficient (R^2^) of 0.99.

The LOD of the UF-qPCR assay was determined by analyzing the replicate serial dilutions of the same QS serum sample (Table 1). The positive rate was calculated by referring different concentration levels. The statistic analysis using PROBIT predicted a LOD of 12.1 IU/ml.

**Table 1:** LOD of the UF-qPCR assay determined by analyzing HBV QS serum samples.

Nominal input HBV DNA(IU/ml)	Replicates number	Number detected	Positive rate (%)
20	20	20	100
10	20	18	90
8	16	11	68.8
5	14	6	42.3
2.5	16	2	12.5

Probit results (95% confidence interval) were as follows: 12.1 IU/ml (10.1 to 16.6).

### Diagnostic accuracy of the UF-qPCR assay

Forty serum samples of low viral load were analyzed using the UF-qPCR and the CAP-CTM V2.0 assay system in parallel. As shown in Fig 5C, correlation analysis demonstrated a significantly positive correlation between these two tests (R^2^ = 0.55, P<0.01). The consistency of these two assays was shown in Fig 5D. Nearly all the differences of the paired viral loads were within the range of the mean difference ± 1.96 SD. The mean value of difference between these two groups was 0.01 (log_10_ IU/ml), with a SD of 0.44 log_10_ IU/ml. The Bland-Altman analysis indicated no significant difference in diagnostic accuracy between these two methods.

## Discussion

Since the viral load of HBV patients varies greatly, qPCR assays with a wide detection range are highly desirable. The ultrasensitive HBV DNA detection assays emerged as this study required. The research has demonstrated that the detection of low level of HBV DNA was essential in order to determine the necessity of antiviral treatment [29]. The improved detection sensitivity of ultrasensitive HBV DNA detection assays is essentially dependent upon an increased sample input volume [30]. Therefore, a large volume of sample is necessary for the preparation of increased amounts of DNA template. The majority of the commercial ultrasensitive assays employ the MBs method [17–20]. Despite the high recovery obtained, these methods are dependent upon costly and specialized equipment. In contrast, UF is a simple and low-cost sample preparation tool. With its advantage in handling large volumes of sample, UF has the potential to be employed as a nucleic acids preparation facility for the ultrasensitive DNA detection assays.

Although the UF filtration membrane can retain the double-stranded DNAs [31], the high level of protein in the serum renders the treatment of the original sample impracticable. The purpose for HBV DNA extraction procedure of this study consists of two major parts: (1) to remove the potential inhibitors of amplification and (2) to isolate and concentrate HBV DNA using UF devices. As heme and immunoglobulin G have been identified as major inhibitors for PCR [32], these proteins should be removed prior to PCR [33,34].This study initially attempted to digest the serum proteins with proteinase K; however, the UF concentrate resulting from the digestion mixture is still highly viscous, indicating proteins were not thoroughly digested into small fragments that can pass through the UF membrane. Next, it was attempted to precipitate the proteins before UF. The serum samples were treated with precipitants including NaOH, SDS-KAc and phenol, and the resultant supernatants were transferred to UF devices. Among these methods, phenol showed the best effect of protein removal. The phenols were known to bind to proteins by forming hydrogen bonds with peptide bond oxygens [35].The OD_260/280_ ratio of the DNA concentrate ranged from 1.7 to 2.0, indicating highly purified DNA was obtained. The amplification efficiency of qPCR with DNA prepared by UF was approximately 90%, demonstrating the UF-based DNA extraction is compatible with qPCR.

The retention of DNA by UF is majorly affected by DNA size and pore size of filtration membrane [36]. As reported, the plasmid DNA (3.0 to 17 kbp) transmission significantly decreased with increasing pore size of the membrane, which indicated that the pore size (namely NWML) played a key role in affecting DNA recovery [37]. In this work, HBV DNA recoveries obtained from UF extractions were above 80% in all groups, indicating HBV DNA can be effectively retained by UF devices with NWML<100K. The UF extraction is able to achieve a DNA recovery comparable to the MBs method, without the necessity of specialized instrument. In comparison with the DNA-extraction column method that using the principle of DNA adsorption onto silica surfaces, UF DNA extraction based on size selected retention is favorable over the column method with regard to DNA recovery in handling the low abundant DNA samples.

According to the manufacture’s instruction, the recovery rate of 1159 bp dsDNA using the 100K filter device was higher than 90%. However, recovery of the 3.2 kb HBV DNA determined in this work was only ~80%, indicating a certain proportion of DNA was lost during the DNA preparation step. The loss of DNA can be ascribed to two reasons: (1) The detainment of DNA in phenol phase. As has been reported, about 10% of the DNA is detained in phenol phase in the protein precipitation step [38]; (2) Adsorption of DNA to the filtration membrane. It was found that the DNA recoveries obtained from high-titer samples were slightly higher than those of low-titer samples, indicating a certain amount of DNA was adsorbed on the filtration membrane. This explanation was also supported by results of the solution depletion test, which showed that about 1ng HBV DNA was retained on the membrane.

From the results of performance evaluations, it appeared that the UF-qPCR assay was highly sensitive. The LOD was determined to be 12.1 IU/ml, which was compared favorably to other ultrasensitive qPCR assays reported. The linearity was demonstrated between 25 and 2.5×10^8^ IU/ml, with a slope of 0.95 and R^2^ values of 0.99, indicating the reliability of the DNA preparation method. The precision of the UF-qPCR assay was comparable to the CAP-CTM V2.0 assay [39].The Bland-Altman analysis also indicated a good correlation between these two methods. These results provide evidence that the performances of these two assays are comparable in analyzing serum samples of different viral loads.

As shown in Table 2, the UF-qPCR was compared with the CAP-CTM V2.0 assay system. These two methods yielded comparable dynamic range. The CAP-CTM method relies on specialized equipment and is advantageous in handling a large batch of samples, while the UF-qPCR is suitable for handling a small or medium quantity of samples with manual operation. In comparison with CAP-CTM V2.0, the UF-qPCR is advantageous with regard to sample/reagent consumption. The analysis time needed for UF-qPCR is also shorter than CAP-CTM V2.0 (3h vs 5h). The major advantage of the UF-qPCR method is low cost. As compared to the CAP-CTM V2.0 assay system, instrument and running costs using the UF-qPCR assay are apparently lower.

**Table 2:** Comparison of the UF-qPCR and the CAP-CTM V2.0 method.

Characteristics	UF-qPCR	CAP-CTM V2.0
Serum volume input (μl)	250	500
DNA concentrate /total reaction volume (μl)	10/30	50/100
Dynamic range (IU/ml)	12–1.0×10^8^	20–1.7×10^8^
LOD	12	20
Additional equipment needed/ equipment cost (RMB, Yuan)	No/None	Yes/~2 million
Running cost (RMB, Yuan)	50–60	300–350
Time-consuming	3–3.5 h (24 samples) Extraction:~2 h Amplification:1.5 h (40 cycles)	~5 h (24–72 samples a time) Extraction:~2.5 h Amplification: 2.5 h (60 cycles)

In conclusion, the UF-qPCR assay is reliable, highly sensitive, affordable and time-saving. The method can be used for the ultrasensitive detection of serum HBV. This study finds it to be a powerful tool to achieve optimal monitoring of antiviral therapy and timely treatment adaptation. In addition, with its advantage in handling large volumes of sample, UF based extraction method hold the potential to be employed as a nucleic acids preparation facility especially for dealing with various types of samples with low nucleic acids content.

## Supporting information

S1 Fig Standard curve established for HBV DNA quantitation. Serial dilutions of a QS serum sample were used as standards. The standard curve was established by plotting logarithmic HBV concentrations to Ct values.
(TIF)
